# The Effects of Pulsed Electromagnetic Field (PEMF) on Muscular Strength, Functional Performance and Depressive Symptoms in Elderly Adults with Sarcopenia: A Short-Term Intervention

**DOI:** 10.3390/life15071111

**Published:** 2025-07-16

**Authors:** Patrícia Sardinha Leonardo, Alberto Souza Sá Filho, Pedro Augusto Inacio, Paulo Ricardo França, Vicente Aprigliano, Fernando Teixeira, Michel Monteiro Macedo, Douglas Farias Fonseca, Pedro Sardinha Leonardo Lopes-Martins, Gustavo De Conti Teixeira Costa, Rodrigo Alvaro Brandão Lopes-Martins

**Affiliations:** 1 Faculty of Medicine, Redentor University Center—AFYA, Avenida Presidente Dutra, nº 1155—Cidade Nova, Itaperuna 28300-000, RJ, Brazil; patricia.sardinha@uniredentor.edu.br (P.S.L.); paullofranca6@gmail.com (P.R.F.); michel.macedo@uniredentor.edu.br (M.M.M.); pdlopesmartins@gmail.com (P.S.L.L.-M.); rodrigo.martins@uniredentor.edu.br (R.A.B.L.-M.); 2 Graduate Program, Evangelical University of Goiás (UniEVANGÉLICA), Anápolis 75083-515, GO, Brazil; alberto.filho@unievangelica.edu.br (A.S.S.F.); pedroqinacio@gmail.com (P.A.I.); fernando@terascience.com.br (F.T.); douglas_bg_2009@hotmail.com (D.F.F.); 3 Escuela de Ingeniería de Construcción y Transporte, Pontificia Universidad Católica de Valparaíso, Avda Brasil 2147, Valparaíso 2362804, Chile; 4 Faculdade de Educação Física e Dança—FEFD, Universidade Federal de Goiás (UFG—Campus Samambaia), Goiânia 74690-900, GO, Brazil; conti02@ufg.br; 5 Hospital do Câncer de Muriaé da Fundação Cristiano Varella, Fundação Cristiano Varella, Av. Cristiano Ferreira Varella, 555—Bairro Universitário, Muriaé 36888-233, MG, Brazil

**Keywords:** aging, muscle function, Pulsed Electromagnetic Field therapy, PEMF

## Abstract

Despite the benefits of resistance training in mitigating sarcopenia, adherence among frail older adults is often limited by osteoarticular pain, comorbidities, and logistical barriers. Pulsed electromagnetic field (PEMF) therapy has emerged as a potential alternative. However, evidence regarding its effects on functional and psychological parameters remains scarce. **Objectives:** To assess the effects of 12 PEMF therapy sessions on knee extensor strength and functional performance (Timed Up and Go test—TUG) in older adults with sarcopenia. Secondary outcomes included changes in calf circumference (CC), SARC-F + CC scores, and depressive symptoms. **Methods:** A controlled, non-randomized experimental design was employed, with a pre-intervention control group serving as a baseline reference (PEMF group: n = 25; control group: n = 16). Participants received 12 PEMF therapy sessions (three times per week) targeting the quadriceps and gastrocnemius muscles. Outcomes were measured using knee-extension dynamometry, TUG, CC, SARC-F + CC, and the Yesavage Geriatric Depression Scale. Statistical analyses included ANCOVA, with baseline values as covariates. **Results:** Significant improvements were observed in knee-extension strength, which increased from 13.05 ± 4.8 kgf to 18.56 ± 8 kgf (*p* < 0.001); TUG test time improved from 23.1 ± 14.4 to 18.7 ± 10 s (*p* = 0.048); SARC-F + CC scores decreased from 11.6 ± 8.2 to 6.5 ± 7.6 (*p* < 0.001), though the interaction effect with time was not significant (*p* = 0.252). No statistically significant changes were observed in CC, which increased from 34.0 ± 4.0 cm to 36.0 ± 3.9 cm following the intervention (*p* = 0.548). Yesavage Geriatric Depression Scale scores improved significantly (7.9 ± 2.4 to 5.4 ± 1.7, *p* = 0.0013). **Conclusions:** PEMF therapy significantly improved lower-limb muscle strength and functional mobility in elderly individuals with sarcopenia. Additionally, depressive symptoms were significantly reduced. However, no significant changes were observed in CC or SARC-F + CC.

## 1. Introduction

It is estimated that the global prevalence of sarcopenia ranges from 10% to 16% in healthy older adults and may reach up to 76% in hospitalized patients, significantly increasing the risk of falls, fractures, and premature mortality [1,2,3,4,5]. Beyond its functional impact, sarcopenia is associated with prolonged hospitalization, early institutionalization, and a substantial increase in public healthcare costs, reinforcing the need for effective and accessible interventions [6,7,8].

To date, physical exercise remains the most effective treatment for sarcopenia in the elderly [9,10,11]. Randomized controlled trials have demonstrated positive effects of resistance training on muscle mass, strength, and physical performance [10,11,12,13,14]. However, adherence among frail older adults is often limited by factors such as osteoarticular pain, comorbidities, and logistical barriers, necessitating the search for alternative strategies for this population [4,11,15]. In this regard, emerging technological innovations, such as pulsed electromagnetic field (PEMF) therapy, may offer significant advancements in addressing this condition [16,17,18,19,20]. PEMF, which operates based on the principle of electromagnetic induction, utilizes alternating magnetic fields to generate electrical currents that depolarize neuromuscular tissue, resulting in both pulsatile and tetanic contractions. PEMF induces muscle contractions that occur independently of the brain’s central command, through the direct depolarization of motor neurons, leading to a substantial calcium influx [18,19,21].

This stimulation appears to induce neuromuscular adaptations similar to those elicited by resistance training [16,22] without the need for external mechanical load, making it a potentially viable alternative for older adults with locomotor limitations. Pilot studies suggest that PEMF application may improve muscle strength and mobility in the elderly [17,23]; however, evidence remains scarce, particularly regarding its effects on functional and psychosocial parameters, such as depressive symptoms, which frequently accompany functional decline in this population.

In this context, the primary objective of the present study was to evaluate the effects of 12 PEMF sessions on muscle strength (in the knee extensors) and functional performance (Timed Up and Go test—TUG) in older adults with sarcopenia. Secondarily, changes in calf circumference (CC), SARC-F + CC scores, and depressive symptoms were analyzed. Two main hypotheses were tested: (H^1^) that PEMF application would result in significant gains in muscle strength and functionality compared to baseline and (H^2^) that the PEMF intervention would lead to maintenance of the CC, reductions in SARC-F scale scores, and reductions in depressive symptoms.

## 2. Methods

### 2.1. Experimental Approach

This study followed a controlled and non-randomized experimental design with pre-intervention adjustments in which a pre-intervention control group was included to account for potential baseline variations. The experimental group (PEMF: n = 25) underwent the PEMF intervention, while the control group (CON: n = 16) was assessed only at baseline to serve as a reference for initial conditions. To enhance the robustness of the analysis, ANCOVA was applied, using baseline values as covariates, ensuring that the observed effects of PEMF were primarily attributable to the intervention rather than to random fluctuations over time.

To minimize bias in the absence of randomization, an independent control group was assessed prior to the intervention period. This group was recruited separately, after all data collection from the PEMF group had been completed, thereby avoiding any temporal overlap or procedural contamination. Control participants were evaluated only once, using the same evaluators, protocols, and equipment as were used for the PEMF group, and did not receive any intervention. This pre-intervention control group served as a reference for baseline conditions, allowing statistical adjustment in the ANCOVA models. The purpose of this structure was to isolate the effects of the PEMF intervention while minimizing expectancy bias and reducing the influence of natural fluctuations over time. By including the control group only at baseline, we ensured independence from treatment exposure, and thus, any changes observed in the PEMF group could be interpreted with greater internal validity.

The study was conducted following the guidelines for reporting parallel-group randomized trials, as outlined in the CONSORT (Consolidated Standards of Reporting Trials) Statement (https://www.equator-network.org/reporting-guidelines/consort/, accessed on 30 January 2025). The study adhered to Resolution 466/2012 of the National Health Council and received approval from the Research Ethics Committee (CAAE: 63398622.8.0000.5076; No. 58.33815). Participants were recruited via a public call within an ongoing extension project at the university. All participants reviewed and signed an informed consent form that provided detailed information about the experimental procedures, as well as potential risks and discomforts. This information was also explained verbally in detail. Following the selection process, acceptance, and the participants’ indicating their understanding of the risks associated with physical exercise, all participants provided their written consent.

### 2.2. Participants and Study Design

Twenty-five elderly people representing both sexes (10 men and 15 women) were recruited in a first public call for the PEMF group. For the control group (CON), a new recruitment call was conducted, enrolling sixteen older adults of both sexes (6 men and 10 women). Participants in the CON group completed the assessment sessions and were not provided with training. Inclusion criteria were defined as follows: (1) age ≥ 65 years; (2) ability to ambulate independently without walking aids; (3) preserved cognitive status and autonomy in performing basic activities of daily living; and (4) sufficient lower-limb motor function, defined as the ability to voluntarily perform active knee extension against gravity or minimal resistance, as evaluated during the screening session. Exclusion criteria included severe physical or mental comorbidities that impaired the individual’s ability to perform physical exercise. Sample selection occurred between June 2023 and January 2024. Participants’ identities were kept confidential, and they were identified by letters corresponding to the initials of their first and last names, excluding “junior”, “son”, and “grandson”, followed by their date of birth and gender (M or F), ex., WA120568M.

Sarcopenia classification in the present study was based on the updated criteria proposed by the European Working Group on Sarcopenia in Older People, which emphasizes low muscle strength as the primary indicator of sarcopenia. Participants were screened using the SARC-F questionnaire in combination with CC (SARC-F + CC), a tool validated for use in primary care. A SARC-F score ≥ 4 combined with CC < 31 cm was considered to indicate probable sarcopenia. Additionally, all included participants demonstrated impaired functional performance on the TUG test, further supporting the classification of sarcopenia according to the EWGSOP2 definition [24].

Participants were recruited through a public call in conjunction with a continuous-flow project at the university. They completed an evaluation form with personal data, signed the informed consent form, and underwent initial screening tests during their first visit. The initial test battery included the following: (a) a body-composition assessment using bioimpedance; (b) the SARC-F questionnaire and calf perimetry; (c) functionality tests (Timed Up and Go); (d) lower-limb strength testing; (f) psychometric questionnaires on depressive and cognitive symptoms. Based on the initial screening, participants were then guided to undergo the PEMF intervention. Table 1 presents the anthropometric and body-composition characteristics of both the PEMF and CON groups.

### 2.3. PEMF Therapy Protocol

Participants underwent 12 sessions of PEMF therapy over a period of four weeks, with three sessions per week (e.g., Monday, Wednesday, and Friday) and intervals of 48 h between sessions. Each session lasted 30 min. The total equipment load per handle was 7.5 Teslas. Four handles were used in total (two concave and two flat). The two flat handles were positioned over the thighs, and the two concave handles were positioned over the calves. For the positioning of the flat handles on the thighs, the muscular belly of the vastus lateralis was used as a reference point. For the positioning of the concave handles on the calf, the point of greatest circumference was used as a reference point. Both anatomical points were identified in the participants by visual inspection. The participants remained lying on a stretcher throughout the intervention.

The first two sessions were used to adapt participants to the electromagnetic field stimulus. Submaximal contractions were applied for 30 min with a frequency of 10 Hz, with 5 s of pulsating contraction and 5 s of pause. An intensity of 10–20% (0.75 to 1.5 Teslas) was used for adaptation, and this value could be increased by another 10%, depending on the participant’s tolerance for the stimulus.

**Figure 1:**
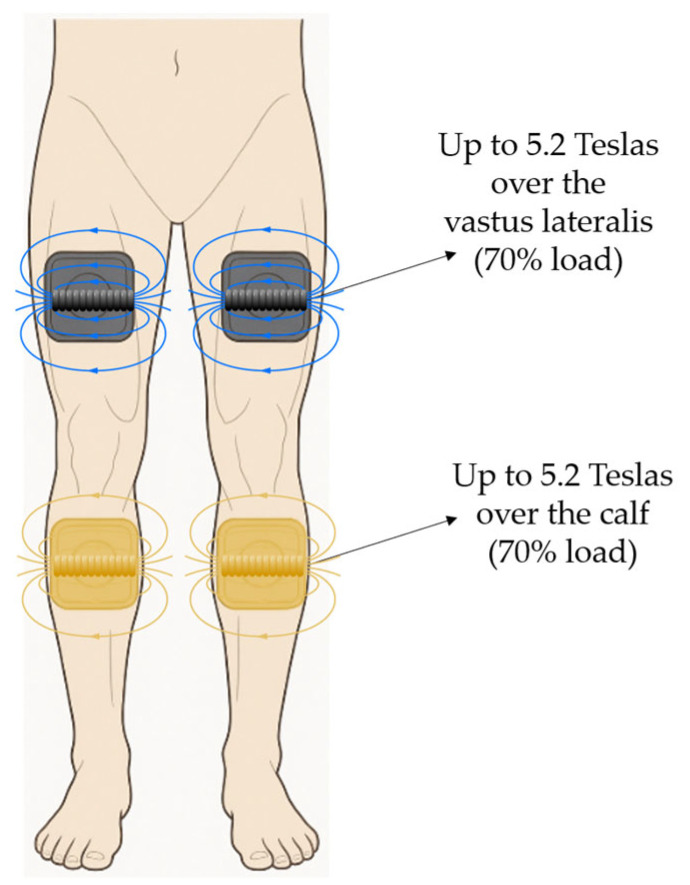
Application of PEMF and handle location. Illustration of the intervention setup used in the present study. The participant is positioned in the supine posture with four PEMF applicators (two per limb) placed bilaterally on the anterior thigh (vastus lateralis region) and posterior leg (gastrocnemius region). This anatomical distribution was standardized for all participants.

In the subsequent 10 sessions, cycles of submaximal contractions (5 Hz, 5 s) and sustained tetanic contraction (30 Hz, 5–8 s) were employed. The elderly participants started the sessions at 40% intensity (3.0 Teslas) and progressed by another 10% per session up to a limit of 70% intensity (5.2 Teslas). All participants completed the allotted 30 min session. The choice of PEMF parameters in the present study was based on previous literature reporting effective therapeutic windows. Commonly, studies have employed intensities between 1.0 and 7.0 Teslas and frequencies from 5 to 50 Hz, with session durations ranging from 20 to 30 min [16,17,22]. Figure 1 shows the PEMF equipment and the positioning of the handles.

### 2.4. Sample Characterization

Sample characterization involved measuring body mass (kg), height (m), lean body mass (kg), and body fat mass (kg). Body mass index (BMI) was calculated from body mass and height. Bioelectrical-impedance analysis was performed using tetrapolar equipment (BIA, Tera Science, São José dos Campos, SP, Brazil), with data transmission to a cloud system available at https://www.terascience.com.br. Preparation criteria for bioimpedance testing included the following: fasting for at least 4 h before the test, avoiding strenuous physical activity within 24 h prior to the test, urinating at least 30 min before the exam, refraining from alcohol consumption within 48 h prior, and avoiding diuretic use for 7 days prior to the test.

### 2.5. Outcome Variables

The primary outcomes were defined based on muscle strength and structural and functional measures, including the following: (a) knee extension and (b) functional mobility assessed using the TUG test. The secondary outcomes were (a) CC measurement; (b) SARC-F + CC questionnaire score; and (c) Yesavage Geriatric Depression Scale (GDS-15) score.

### 2.6. Primary Outcome Measures

#### 2.6.1. Evaluation of Lower-Limb Muscle Strength

Lower-limb muscle strength was evaluated using the E-lastic™ Computerized Dynamometry System. This system, based on a load cell, assesses muscle force, speed, and movement acceleration. The system transmits real-time data to mobile devices via Bluetooth and can also transmit remotely via the internet. For the assessment of knee-extension strength, participants remained seated with their knees flexed between 70° and 90° (with little freedom of movement) and were required to perform maximal knee extension, sustaining the contraction for three seconds. The evaluators provided verbal stimulation to ensure maximum effort.

Three maximum-strength contractions were performed for each limb. Only the right side was used for analysis. The highest force value from the three attempts was recorded. The system uses adjustable wristbands, ensuring there was no discomfort other than that resulting from the effort exerted by the participant.

#### 2.6.2. Timed Up and Go Test (TUG)

The TUG test is a widely used tool for assessing fall risk in the elderly [1]. Its validity and reliability have been previously tested [25]. It measures the time required for a person to stand from a chair, walk 3 m, turn around, return to the chair, and sit down. For the TUG test, an order was issued by the evaluators, “I am going to ask you to stand up from your chair, walk to the 3-m mark, turn around, walk back and sit down again as quickly as possible, but do not run.” Time recording began when the participant stood up and stopped when they sat down again. The TUG results were categorized as follows: (a) up to 10 s (normal performance for healthy adults, low fall risk); (b) between 11 and 20 s (normal for frail or debilitated elderly individuals, but still independent in most daily activities, low fall risk); (c) between 21 and 29 s (functional assessment required, moderate fall risk, specific fall-prevention measures recommended); (d) ≥30 s (functional assessment required, high fall risk, specific fall-prevention measures recommended).

To ensure consistency and minimize the influence of test learning, each participant performed two trials consecutively during the same session. The first trial served as familiarization, following standard protocol, and was not used for analysis. The second trial was used for data collection and statistical analysis. A rest period of approximately one minute was provided between trials to reduce fatigue. Verbal encouragement and instructions were standardized by the same evaluator across all participants to ensure procedural uniformity.

### 2.7. Secondary Outcome Measures

#### 2.7.1. Sarcopenia Assessment and Calf Perimetry

The adapted SARC-F sarcopenia-screening questionnaire with CC (SARC-F + CC) is a validated screening tool for sarcopenia risk in the elderly [24,26,27]. The questionnaire comprises five questions assessing strength, walking ability, ability to get up from a chair, ability to climb stairs, and history of falls. Each question is rated on a scale from 0 to 2 points, for a total score ranging from 0 to 10. A score of 4 or higher indicates a risk of sarcopenia. SARC-F has an excellent specificity (85%) and a negative predictive value of 96% [27].

Calf perimetry, an essential parameter in sarcopenia diagnosis [28,29], was measured according to the guidelines set by the International Society for the Advancement of Kinanthropometry (ISAK). The largest circumference of the calf, incorporating the gastrocnemius muscle, was determined for each participant. The measurement was carried out pre- and post-intervention.

#### 2.7.2. Yesavage Depression Scale

The Yesavage scale is a widely used instrument for the assessment of depression in the elderly. It consists of 15 yes/no questions addressing cognitive, affective, and behavioral aspects of depression. Scores range from 0 to 15, with values ≥ 6 indicating possible depression and values ≥10 indicating probable depression [30]. The scale has been previously validated for the elderly Brazilian population, demonstrating good sensitivity and specificity in detecting depressive symptoms, and is widely used in clinical and research settings [31]. The Yesavage scale was applied by a trained evaluator at the end of the initial visit and in the post-intervention period.

### 2.8. Size of Study

Sample-size calculation was performed using G*Power software (version 3.1) and was based on the difference between two dependent means [32]. The data were analyzed using F tests in repeated-measures ANOVA with a within–between interaction. The input measures for the a priori computation of the required sample size were as follows: effect size *f* = 0.35; α err prob = 0.05; power (1-β err prob) = 0.85; number of groups = 2; number of measurements = 2; corr among rep measures = 0; nonsphericity correction ε = 1. The output was as follows: noncentrality parameter λ = 9.8; critical F = 4.0981717; numerator df = 1.0; denominator df = 38.0; total sample size = 40; actual power = 0.862.

### 2.9. Bias

To minimize potential biases in this study, an independent pre-intervention control group was included to account for external factors that could influence participants’ responses over time. This structure allowed for an assessment of whether the observed effects of PEMF were truly associated with the intervention or whether they could be attributed to natural or random variations in physiological and functional measures. Additionally, statistical analyses were conducted with adjustments for this variable as a covariate, enhancing the robustness of the analyses and their sensitivity to the effects of PEMF.

The use of standardized procedures for data collection, conducted by the same evaluator, further reinforced the reliability of the measurements, minimizing potential distortions in the interpretation of results. Additionally, the principal investigator was blinded to data analysis.

Despite these measures, the absence of a post-intervention measurement of the control group remains a limitation that may introduce potential biases, which should be considered in the interpretation of the findings.

### 2.10. Statistics

Data were analyzed descriptively and are presented as mean ± SD. Normality tests were conducted (Shapiro–Wilk). A one-way ANOVA was used to compare the baseline data of the elderly participants, with the data stratified by SARC-F + CC scores. An analysis of covariance (ANCOVA) was conducted to compare the means of the dependent variables across time, adjusting for the influence of the independent control group. The effect size was calculated using partial Eta squared (η^2^p). The 95% confidence intervals (95% CI) of all dependent variables were determined. Statistical significance was set at *p* = 0.05. All analyses were performed using SPSS 20.0 (Chicago, IL, USA), and graphical representations were created using GraphPad Prism software (v.8, Boston, MA, USA).

## 3. Results

### 3.1. General Information

Per the analysis of the assumptions of normal distribution by the Shapiro–Wilk test, the variables age (*p* = 0.539), lower-limb strength (*p* = 0.451), TUG (*p* = 0.618), and CC (*p* = 0.347) followed normal distributions.

Participants were stratified into two subgroups based on SARC-F + CC scores (<10 and ≥10) to explore whether baseline functional status influenced the magnitude of response to PEMF; however, no significant differences were observed between the subgroups. The participants’ data were stratified based on the SARC-F + CC scores and then characterized; these data are presented in Table 2 as descriptive statistics. The values are expressed as mean ± SD, providing an overview of the morphological and performance characteristics of the participants. The data in Table 2 did not show significant differences for almost any baseline measure, except for CC in the control group (*p* = 0.004). Therefore, the data were analyzed collectively (PEMF: n = 25 vs. CON: n = 16).

#### Adverse Effects and Treatment Adherence

During the PEMF-therapy intervention period, no severe adverse events were reported by the participants. All 25 individuals completed the 12 treatment sessions without significant complications. Participants were monitored for possible side effects throughout the study and after its conclusion. Participants were instructed to report any discomfort or unexpected symptoms.

Nevertheless, mild events were recorded during the initial phase of treatment (n = 6; 24%), including transient sensations of muscle discomfort, which required a temporary reduction in the intensity of the electromagnetic field. However, these effects were limited to the initial sessions and did not necessitate treatment interruption. No participant reported persistent pain, adverse cardiovascular effects, or deterioration in functional status following the intervention.

Adherence to the protocol was complete, and no participant requested the suspension of sessions due to discomfort or deleterious effects. PEMF therapy was well tolerated and demonstrated a favorable safety profile for elderly individuals with sarcopenia.

### 3.2. Primary Outcomes

#### 3.2.1. Knee-Extension Dynamometry

**Figure 2:**
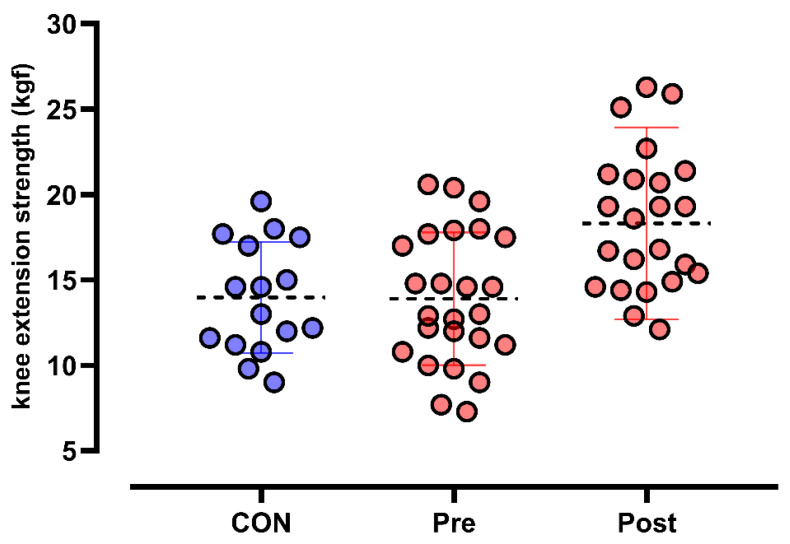
Effects of PEMF treatment on muscle strength in elderly individuals.

An ANCOVA was conducted to evaluate the effects of time and the time × group interaction on lower-limb strength (kgf), with the pre-experimental condition of the control group as a covariate. The results indicated a significant main effect of time (F_1,14_ = 17.248, *p* < 0.001; η^2^p = 0.552), demonstrating a significant change in lower-limb strength over time, regardless of the group. Additionally, a significant interaction effect between time and group was identified (F_1,14_ = 12.318, *p* = 0.003; η^2^p = 0.468), suggesting that the degree of increase in lower-limb strength over time differed between groups. The residual error was 280.780, reinforcing the robustness of the analysis. These findings indicate that even after controlling for the baseline condition of the control group, the groups exhibited distinct responses to the intervention, highlighting the influence of the treatment on lower-limb muscle strength. The η^2^p suggests that the time factor accounted for approximately 55.2% of the total variance, while the time × group interaction explained 46.8% of the variance in lower-limb strength. Figure 2 presents the dynamometry data for knee extension.

#### 3.2.2. TUG Test

**Figure 3:**
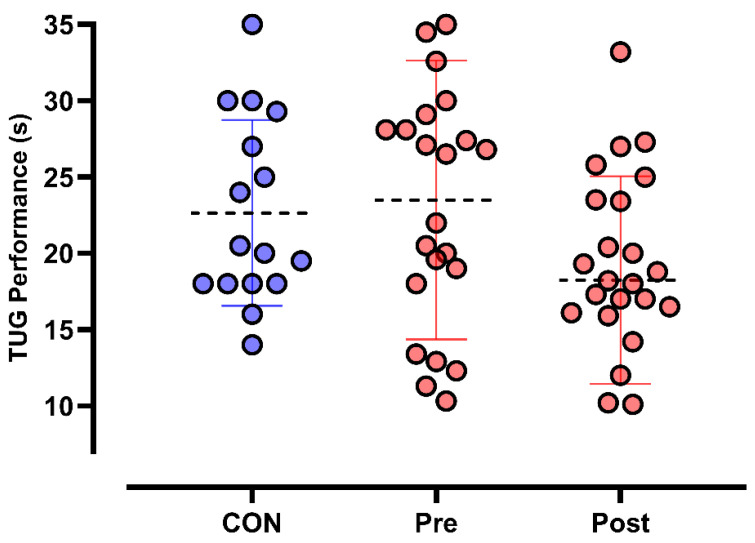
Timed Up and Go (TUG) test performance pre- and post-PEMF treatment.

The comparison of TUG means, controlling for the pre-experimental condition of the control group as a covariate, indicated a significant main effect of time (F_1,14_ = 4.711, *p* = 0.048; η^2^p = 0.252), suggesting a statistically significant change in TUG performance over time. Additionally, the interaction between time and group was also significant (F_1,14_ = 7.521, *p* = 0.016; η^2^p = 0.349), indicating that the change in TUG performance differed between groups over the analyzed period. These findings suggest that the intervention had a significant impact on improving TUG performance over time. The η^2^p value suggests that time accounted for approximately 25.2% of the total variance, while the time × group interaction explained 34.9% of the variance in TUG performance. Figure 3 presents the values of TUG performance.

### 3.3. Secondary Outcomes

#### 3.3.1. Calf Circumference

The ANCOVA, controlling for the pre-experimental condition of the control group as a covariate, indicated that the main effect of time was not significant (F_1,14_ = 0.378, *p* = 0.548; η^2^p = 0.024), suggesting that there was no statistically significant change in CC over time (CON: 31.0 ± 5.1 cm; PEMF pre: 34.0 ± 4.0; PEMF post: 36.0 ± 3.9 cm). Similarly, the interaction between time and group was also not significant (F_1,14_ = 0.178, *p* = 0.679; η^2^p = 0.038), indicating that the progression of CC did not differ between groups over the analyzed period. The residual error was 115.664, highlighting the sample variability. The magnitude of the effects, represented by the η^2^p, suggests that both time and the time × group interaction accounted for a very small proportion of the total observed variance, reinforcing the finding that the intervention had no significant effects.

#### 3.3.2. The SARC-F + CC Scale

**Figure 4:**
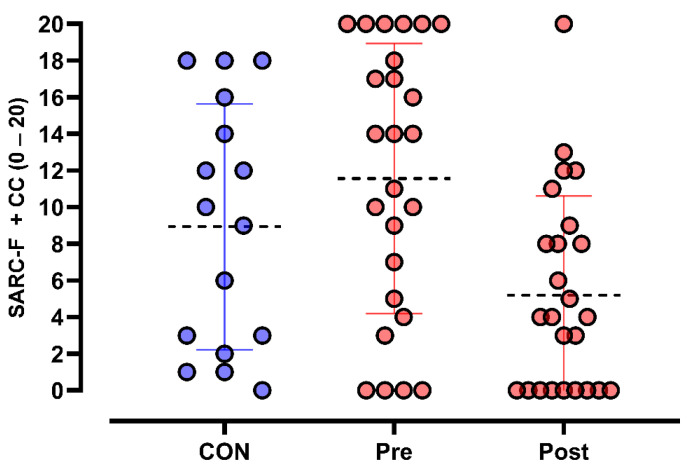
The effects of PEMF on SARC-F + CC scale scores.

The ANCOVA, controlling for the pre-experimental condition of the control group as a covariate, indicated a significant main effect of time (F_1,14_ = 20.568, *p* < 0.001, η^2^p = 0.595), suggesting a statistically significant change in the variable over time. However, the interaction between time and group was not significant (F_1,14_ = 1.427, *p* = 0.252, η^2^p = 0.093), indicating that the progression of sarcopenia did not differ between groups over the analyzed period (CON: 8.9 ± 7.7; PEMF Pre: 11.6 ± 7.4 to Post: 5.2 ± 5.4). These findings suggest that although there was a significant difference between the pre- and post-intervention conditions, this change was similar across groups; this result suggests that the intervention may have influenced the assessed variable, though without substantial differences between the analyzed groups. Figure 4 shows the effects of PEMF on SARC-F + CC scale scores.

#### 3.3.3. Yesavage Depression Scale

**Figure 5:**
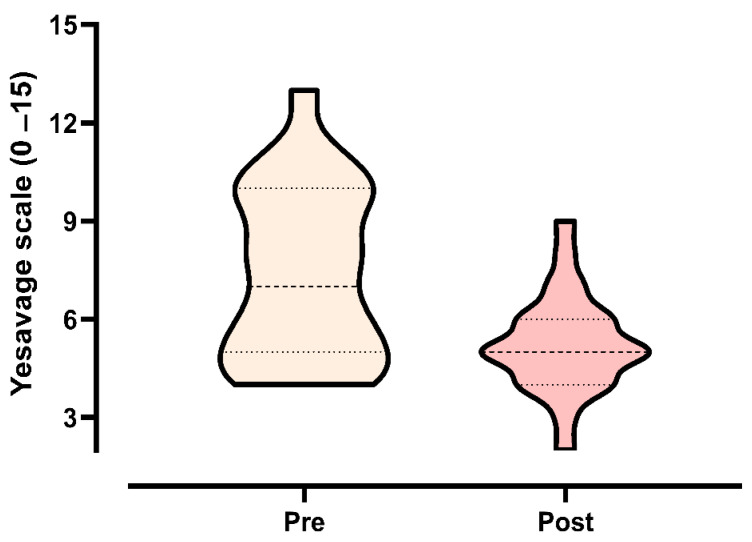
Effects of PEMF treatment pre- and post-intervention.

The Yesavage Geriatric Depression Scale was used to assess depressive symptoms in elderly individuals pre- and post-PEMF treatment. PEMF treatment led to a significant reduction in the individual scores (7.9 ± 2.4 to 5.4 ± 1.7), indicating an improvement in the depressive state (*p* = 0.0013). Figure 5 shows the effects of PEMF treatment pre- and post-intervention.

## 4. Discussion

### 4.1. General Approach

The objective of this study were to evaluate the effects of 12 sessions of treatment with PEMF on strength, functional performance and aspects of muscle structure related to sarcopenia in the elderly. To the best of our knowledge, this is one of the first short-term controlled interventions investigating the effects of PEMF therapy on neuromuscular and psychosocial outcomes in elderly individuals with sarcopenia. Our primary hypothesis (H^1^) was partially supported, since we could not clearly conclude that there was an improvement in some of the parameters analyzed (CC and SARC-F + CC). Despite this, these findings represent a significant advancement in the treatment of sarcopenia in elderly populations, as strength is associated with a positive prognosis related to health and the risk of all-cause mortality. Additionally, the results of the TUG performance test showed a significant improvement, potentially reflecting the primary effects of PEMF on strength. This technology enables muscle stimulation without requiring extensive mobilization, thus generating positive outcomes for elderly sarcopenic individuals while helping to overcome barriers associated with interventions based on physical exercise.

### 4.2. Primary Outcome

Sarcopenia is an increasingly prevalent public health issue that is often overlooked by health systems worldwide [2,3,4]. The progressive loss of muscle mass due to the normal aging process, or as a result of hospitalization and comorbidities, can lead to severe physical disabilities and psychological distress [4,33]. Resistance exercise and improved nutritional intake are established interventions for mitigating sarcopenia [34]. The international guidelines on sarcopenia strongly recommend resistance-based training and suggest increasing protein and caloric intake, with protein supplementation as necessary [34]. Resistance training is well supported by evidence for improving functional outcomes [12,35], although the evidence for its impact on body composition is weaker [35]. A recent multicenter randomized controlled trial demonstrated that a multicomponent intervention comprising moderate-intensity exercise and individualized nutritional counseling significantly reduced (by 22%) the incidence of mobility disability in patients with frailty and sarcopenia [13]. The results of a meta-analysis by Chen et al. [10] align with these findings, demonstrating that moderate-intensity resistance training provided significant benefits for sarcopenic older adults, improving leg-press strength, appendicular skeletal-muscle index, gait speed, and performance on the TUG test (mean difference = −1.74 [95% CI = −3.34 to −0.56]).

The importance of muscle-strengthening activities for mitigating sarcopenia risk has been highlighted in recent literature. Veen et al. [14] demonstrated that adherence to guidelines on muscle-strengthening activities, particularly in combination with aerobic exercise, significantly lowers sarcopenia risk in older adults by enhancing muscle mass and functional performance. However, adherence to resistance-training programs in frail elderly populations poses significant challenges, particularly due to joint limitations and overall physical frailty [11,15]. PEMF emerges as a promising strategy in this context, especially for elderly individuals who face difficulty maintaining traditional exercise routines [17,18]. Our findings support this perspective, as the significant improvements observed, particularly in muscle strength and functional performance, demonstrate the potential of PEMF as an effective intervention. According to the European consensus on sarcopenia, strength measurement is a simple and low-cost method that predicts poor outcomes such as prolonged hospital stays, functional limitations, reduced quality of life, and mortality [4,24].

In our study, knee-extension strength improved significantly after PEMF treatment, potentially impacting performance on functional tasks. In interpreting our findings, it is important to consider that early improvements in muscle strength following short-term interventions are often attributable to neural and psychological mechanisms rather than to structural muscle changes [36,37]. This is especially true in older adults with sarcopenia or pre-sarcopenia, for whom physiological constraints may delay hypertrophic adaptations [38]. Neural mechanisms such as enhanced motor-unit recruitment, synchronization, reduced antagonist coactivation, and improved neuromuscular efficiency have been consistently reported as primary drivers of strength gains in the early weeks of intervention [36,39]. Moreover, psychological factors such as increased confidence, reduced fear of movement (kinesiophobia), and heightened motivation, can also influence performance outcomes, particularly on functional tests such as TUG. Motivation and expectancy effects can further amplify physical outcomes in short-term trials, especially in the absence of blinding or placebo control [40]. Recent studies have demonstrated also that fall-related self-efficacy is significantly associated with gait parameters, including gait speed and step length, in older adults. For instance, a study by Kamide et al. [41] found that lower fall-related self-efficacy was linked to slower gait speed and shorter step length, which are critical components of functional-mobility assessments like the TUG test. While our data suggest a genuine training response to PEMF, it is plausible that these neural and psychosocial factors played a substantial role in the observed improvements.

Interestingly, Sayer and Cruz-Jentoft recently emphasized a shift in the definition of sarcopenia to one with a greater focus on muscle function, as reflected in various international guidelines [4]. Our study demonstrated that PEMF significantly improved functional outcomes in elderly patients, aligning with this updated perspective. This finding reinforces the effectiveness of PEMF therapy in improving functional mobility in older adults, aligning with the literature that identifies the TUG test as one of the main predictors of fall risk and functional decline in this population. According to Beauchet et al. [42], the cutoff time to distinguish older adults at high risk of falls can vary between 10 and 33 s, depending on the level of frailty in the analyzed sample. Our study presented a pre-intervention mean time of 23.5 ± 9.1 s, which was reduced to 18.2 ± 6.8 s, characterizing the elderly participants in our study as being at potential risk, even in the face of significant improvement. However, studies such as that by Filippin et al. [43] suggest that values above 7.5 s may already indicate sarcopenia and reduced functional capacity in community-dwelling older adults.

In a study by Leonardo et al. [17], sarcopenic older adults underwent 12 sessions of pulsed electromagnetic field (PEMF) therapy over four weeks, with three weekly sessions, using protocols that combined submaximal and supramaximal contractions. The results revealed a significant improvement in performance on the TUG test, with the average execution time decreasing from 40 ± 10 s to 22 ± 6 s after treatment. This reduction represents an approximately 47% gain in functional efficiency, indicating that PEMF therapy may enhance locomotor capacity and postural stability in older adults. Although this study is methodologically similar, the magnitude of the reduction observed in the TUG test was not the same, with a significant 31% variation observed in our study.

The mechanism underlying this improvement may be related to PEMF’s ability to generate intense muscle contractions and thus promote neural adaptations similar to those induced by conventional resistance exercise (as observed in our study) [39]. Furthermore, PEMF does not appear to activate nociceptors in the same way as traditional electrical-current stimulation due to its less invasive action, making the intervention more comfortable for older individuals/people undergoing rehabilitation and increasing patient adherence to the treatment [20]. This characteristic is particularly relevant for elderly populations, who often face barriers to engaging in traditional physical-exercise programs. Additionally, the improvement observed in TUG performance may have direct implications for fall prevention (although we do not directly analyze this issue), as the test evaluates critical mobility components, such as gait speed, dynamic balance, and the ability to perform postural transitions. Studies like that by Ortega-Bastidas et al. [44] suggest that instrumented variations of the TUG (iTUG) can further enhance fall-risk prediction by allowing a segmented analysis of different movement phases, such as initial impulse, turning, and post-march stabilization.

Although the present study did not include a placebo-controlled group, which limits the ability to definitively attribute the observed effects to PEMF alone, we applied ANCOVA with baseline values as covariates to minimize bias and isolate the effects of the intervention. While the direction and magnitude of improvements in knee-extension strength and TUG performance align with known neuromuscular adaptations in older adults, we acknowledge that expectancy or nonspecific effects cannot be entirely excluded. Therefore, these findings should be interpreted as preliminary and hypothesis-generating, underscoring the need for future randomized placebo-controlled trials. In neuromuscular research, it is well established that even short-term interventions, when properly applied, can elicit meaningful functional adaptations in older adults. Cadore et al. [45], for instance, demonstrated that a few weeks of resistance training was sufficient to induce significant gains in muscle strength and functional capacity in frail elderly individuals. These results underscore the responsiveness of neuromuscular parameters to intervention and support the plausibility of the effects observed in our study. Furthermore, we employed objective, performance-based outcome measures and standardized all testing procedures. The use of ANCOVA with baseline values as covariates helped to statistically isolate the intervention effect, enhancing the internal validity of our findings.

Importantly, the magnitude of the effects was supported by the calculated effect sizes. The η^2^p suggests that the time factor accounted for approximately 55.2% of the total variance, while the time × group interaction explained 46.8% of the variance in lower-limb strength. Similarly, for the TUG test, η^2^p indicated that time accounted for approximately 25.2% of the total variance, while the time × group interaction explained 34.9% of the variance in performance. These values reinforce the robustness and relevance of the observed changes. While the contribution of factors such as test familiarization and increased motivation cannot be entirely excluded, the improvements reported here exceed what would typically be expected from those influences alone.

### 4.3. Secondary Outcome

Our second hypothesis (H^2^) posited that PEMF therapy would lead to improvements in sarcopenia-related indices and depressive symptoms in elderly individuals. This hypothesis was partially supported. We observed a statistically significant reduction in depressive symptoms, as measured by the Yesavage Geriatric Depression Scale (from 7.9 ± 2.4 to 5.4 ± 1.7; *p* = 0.0013), indicating a potential mental-health benefit associated with PEMF therapy. Additionally, SARC-F + CC scores decreased substantially (from 11.6 ± 7.4 to 5.2 ± 5.4), suggesting improved functional perception. However, the ANCOVA revealed that the interaction between time and group was not statistically significant (*p* = 0.252), indicating that the observed reduction may not be attributable solely to the intervention. This finding underscores the multifactorial nature of perceived functional status and the need for further placebo-controlled investigations. Regarding calf circumference, although a numerical increase was noted (from 34.0 ± 4.0 to 36.0 ± 3.9 cm), the change was not statistically significant (*p* = 0.548). This result was anticipated, as CC has limited sensitivity for detecting subtle muscle hypertrophy and may be influenced by non-muscle tissues such as subcutaneous fat or fluid retention. We highlight that CC is better suited for use as a screening marker rather than a standalone outcome for intervention efficacy. Overall, while the reduction in depressive symptoms provides promising evidence, the effects on structural and composite measures of sarcopenia remain inconclusive.

Due to the scarcity of studies investigating the effects of PEMF specifically in sarcopenic older adults, direct comparisons with data from the literature are limited. Thus, the present findings should be viewed as exploratory and hypothesis-generating. In this light, we will try to contextualize our findings by inference based on evidence on analogous themes.

For example, let us consider the SARC-F scale, a widely used tool for assessing sarcopenia severity and functional decline [28,46]. Barreto de Lima et al. [27] evaluated the diagnostic performance of SARC-F and SARC-CalF in screening for sarcopenia in older adults from Northern Brazil, indicating that the inclusion of CC improves the sensitivity of the test compared to SARC-F alone. The authors observed that SARC-CalF had a better predictive capacity for sarcopenia, while SARC-F exhibited low sensitivity, which could lead to underdiagnosis. Our study found a statistically significant reduction in scores following PEMF therapy (from 11.6 ± 7.4 to 5.2 ± 5.4). However, our ANCOVA results indicated that the progression did not significantly differ between groups, suggesting that while PEMF may have contributed to functional improvements, we cannot attribute the reduction in SARC-F + CC scores exclusively to the intervention. Given the well-established relationship between muscle function and sarcopenia-related disability [3,4], our findings suggest that PEMF might influence functional status in sarcopenic elderly individuals, but further studies are needed to isolate its direct effects.

Additionally, a recent clinical study involving over 300 volunteers confirmed that CC measurement can serve as a simple, rapid, and cost-effective tool for assessing muscle mass and sarcopenia [47]. However, CC may be useful as an initial screening tool, particularly in resource-limited settings, its isolated use for diagnosing sarcopenia presents substantial limitations. Studies have shown that this measure has low specificity for estimating muscle mass, as it does not differentiate between muscle tissue, subcutaneous fat, and fluid retention, which may compromise diagnostic accuracy [4,8]. Additionally, CC exhibits variable correlation with reference methods such as DXA, computed tomography, and magnetic resonance imaging, making its validity for accurately assessing sarcopenia questionable [48]. Aging further affects the reliability of this measure, as intramuscular fat infiltration can mask muscle-mass reduction, leading to an overestimation of muscle status in older adults [49]. Thus, guidelines such as those from the European Working Group on Sarcopenia in Older People (EWGSOP) [24] recommend a combined approach including the assessment of muscle strength and physical performance in addition to imaging methods, when available, to ensure a more accurate diagnosis [3,4].

In this study, we used CC as an additional marker to assess the outcomes of sarcopenia in the elderly patients. Our results demonstrated improvements in CC following PEMF treatment, with CC values increasing from 34.0 ± 4.0 to 36.0 ± 3.9 cm; however, this change was not significant when the control condition was included as a covariate. Despite this, it is not possible to adequately compare these results with data from the literature, since we are pioneers in using these analyses to assess intervention with PEMF.

Sarcopenia has been implicated in an increased risk of cognitive impairment and depression in aging populations. For example, Lee et al. [50] demonstrated a significant correlation between sarcopenia and the risk of depression in elderly Korean women. Depression is associated with decreased metabolism and serotonin function in the central nervous system, as evidenced by results from both animal models and clinical studies [51]. Physical exercise has the ability to significantly modulate adult neurogenesis, leading to improvements in mood [52,53]. Cellular mechanisms, such as the upregulation of neurogenesis, are considered important regulators of mood following exercise. Recent advances in understanding the molecular mechanisms underlying exercise-regulated neurogenesis have expanded our understanding of brain plasticity under both physiological and pathological conditions [54], enabling better management of various psychiatric disorders [51,55,56]. In our study, we found a significant reduction in depressive symptoms, as measured by the Yesavage scale (from 7.9 ± 2.4 to 5.4 ± 1.7, *p* = 0.0013), aligning with results in the literature. However, it is important to acknowledge that we did not compare these results with a control group, limiting our ability to determine whether these improvements resulted directly from PEMF or from other factors, such as increased social interaction or placebo effects.

A systematic review and meta-analysis conducted by Chang et al. [9] investigated the association between sarcopenia and depression, analyzing 15 observational studies with a total of 33,030 participants. The results showed that individuals with sarcopenia had a significantly higher risk of depression, with a crude odds ratio (OR) of 1.640 (95% CI: 1.247–2.155) and an adjusted OR of 1.821 (95% CI: 1.160–2.859), indicating that this relationship persisted even after adjusting for variables such as age, sex, cognitive performance, and physical-activity levels. Furthermore, studies that included handgrip strength and gait speed in the diagnostic criteria for sarcopenia showed a stronger association with depression than those that assessed muscle mass alone. These findings suggest that sarcopenia may be an independent risk factor for depression, possibly due to mechanisms such as chronic inflammation, dysfunction of the hypothalamic–pituitary–adrenal axis, hormonal declines, and reduced physical activity, which impact both muscle health and emotional well-being.

### 4.4. Limitations

Firstly, the experimental design included a control group only for baseline measurements, without a post-intervention assessment, which prevents a direct comparison of the treated group with a group not exposed to PEMF over time. Although ANCOVA was applied to minimize this bias, the absence of a post-treatment control group reduces the ability to infer causality with greater robustness. Future randomized trials with a double-blind, placebo-controlled design are warranted to validate these findings under more controlled conditions. Additionally, although the sample-size calculation indicated adequate statistical power, the sample size was relatively small (n = 25 in the PEMF group and n = 16 in the baseline control group), which may limit the generalizability of the results. Finally, the findings regarding improvements in depressive symptoms should be interpreted with caution, as there was no control group for direct comparison. While the significant reduction in the Yesavage scale scores is a positive indicator, the possibility of placebo effects or the influence of psychosocial factors, such as increased engagement in activities and socialization, cannot be ruled out.

## 5. Conclusions

In summary, this short-term study suggests that PEMF therapy may promote meaningful improvements in lower-limb muscle strength and functional mobility in older adults with sarcopenia. Additionally, a significant reduction in depressive symptoms was observed following the intervention. However, no significant changes were found in calf circumference, and the reduction in SARC-F + CC scores did not differ significantly between groups after adjustment. These findings should be interpreted with caution due to the study’s exploratory nature, the lack of a sham-controlled group, and the potential influence of psychological and expectancy-related factors.

Given the magnitude and direction of the observed changes, particularly a 42.2% increase in knee-extension strength and a 31.6% reduction in depressive symptoms, it is plausible that PEMF elicited real neuromuscular and psychological effects. Nonetheless, we acknowledge that nonspecific effects such as increased attention and motivation may have contributed to the outcomes. Therefore, the present findings should be viewed as preliminary and hypothesis-generating.

From a clinical standpoint, PEMF remains a promising and low-burden strategy, especially for frail older adults with low adherence or tolerance to conventional exercise. Future randomized, double-blind, sham-controlled trials with larger and more diverse samples are essential to confirm the efficacy, mechanisms, and long-term applicability of PEMF therapy in sarcopenia management.

## Figures and Tables

**Table 1:** Anthropometric and body-composition characteristics.

	Age	Body Mass	Height	BMI	Lean Mass	Fat Mass	SARC-F + CC
	(Years)	(kg)	(m)	(kg·m^−2^)	(kg)	(kg)	(Score)
PEMF							
Mean	76.6	72.1	1.60	31.3	47.4	30.4	11.6
SD	6.2	10.2	8.0	5.0	3.0	9.7	7.4
CON							
Mean	73.8	70.5	1.63	30.4	45.4	32.1	8.9
SD	9.0	9.4	9.5	5.5	5.1	9.0	6.7

SD = standard deviation; BMI = body mass index.

**Table 2:** Sample characterization (n = 41 participants).

	SARC-F + CC < 10 (n = 16)		SARC-F + CC > 10 (n = 9)		*p* Value SARC	*p* Value Group
PEMF (n = 25)	Mean ± SD	95% CI	Mean ± SD	95% CI		
Age (years)	76.8 ± 5.9	(73.7–80.0)	76.0 ± 6.8	(70.7–81.2)	0.992	0.566
TUG (s)	22.6 ± 7.8	(18.5–26.8)	24.9 ± 11.4	(16.0–33.7)	0.912	0.876
CC (cm)	35.1 ± 4.0	(32.9–37.3)	32.0 ± 3.1	(29.6–34.5)	0.288	0.768
Knee Extension (kgf)	13.5 ± 3.8	(11.4–15.6)	14.5 ± 4.0	(11.3 ± 17.6)	0.992	0.993
CON (n = 16)	(n = 8)		(n = 8)			
Age (years)	74.1 ± 8.6	(66.1 ± 82.1)	75.1 ± 9.2	(67.2 ± 82.7)	0.921	0.939
TUG (s)	19.9 ± 6.2	(14.0–25.7)	21.3 ± 5.2	(16.9–25.7)	0.985	0.994
CC (cm)	28.7 ± 5.0 *	(24.0–33.3)	33.4 ± 4.4	(29.7–37.1)	0.991	0.004
Knee Extension (kgf)	13.5 ± 3.3	(10.4–16.5)	13.9 ± 3.4	(11.1–16.8)	0.990	0.993

SARC-F + CC = Adapted SARC-F sarcopenia screening questionnaire with calf circumference (CC); SD = standard deviation; TUG = Timed up and go test; (*) = significant differences between groups (CON vs. PEMF SARC-F + CC < 10).

## Data Availability

All raw data will be available upon a reasonable request to the corresponding author.
